# Seminal Plasma Triggers the Differential Expression of the Glucocorticoid Receptor (*NR3C1/GR*) in the Rabbit Reproductive Tract

**DOI:** 10.3390/ani10112158

**Published:** 2020-11-19

**Authors:** Mateo Ruiz-Conca, Jaume Gardela, Amaia Jauregi-Miguel, Cristina A. Martinez, Heriberto Rodríguez-Martinez, Manel López-Béjar, Manuel Alvarez-Rodriguez

**Affiliations:** 1 Department of Biomedical and Clinical Sciences (BKV), Division of Children’s and Women Health (BKH), Obstetrics and Gynecology, Linköping University, 58185 Linköping, Sweden; mateo.ruiz@uab.cat (M.R.-C.); jaume.gardela@uab.cat (J.G.); cristina.martinez-serrano@liu.se (C.A.M.); heriberto.rodriguez-martinez@liu.se (H.R.-M.); 2 Department of Animal Health and Anatomy, Veterinary Faculty, Universitat Autònoma de Barcelona, 08193 Bellaterra, Spain or manel.lopezbejar@westernu.edu; 3 Department of Biomedical and Clinical Sciences (BKV), Division of Molecular Medicine and Virology (MMV), Linköping University, 58185 Linköping, Sweden; amaya.jauregi.miguel@liu.se; 4 College of Veterinary Medicine, Western University of Health Sciences, Pomona, CA 91766, USA

**Keywords:** glucocorticoid receptor, gene expression, RT-qPCR, seminal plasma, female genital tract, rabbit

## Abstract

**Simple Summary:**

Glucocorticoids are steroid hormones modulating different functions in mammals, including reproduction, that act through the glucocorticoid receptor, encoded by the gene called *NR3C1*. Here, we describe how the expression levels of the glucocorticoid receptor change along the different compartments of the female rabbit internal reproductive tract 20 h after insemination with sperm-free seminal plasma or natural mating (whole semen) (*Experiment 1*) and how these levels change at 10, 24, 36, 68, and 72 h post-mating, during specific stages over time, i.e., ovulation, fertilization and the interval of early embryo development to the morula stage occurs (*Experiment 2*). *NR3C1*-upregulation was found in the infundibulum at 20 h after all treatments, especially after sperm-free seminal plasma infusion compared to mating (*Experiment 1*). In *Experiment 2*, the receptor gene expression levels increased in a spatio-temporal sequence, corresponding to the assumed location of the rabbit embryos (particularly morulae) in the oviductal various segments and timepoints (particularly 72 h), compared to down-expression at uterine regions. We conclude that *NR3C1* may play a relevant role in the rabbit female reproductive tract.

**Abstract:**

Rabbits are interesting as research animal models for reproduction, due to their condition of species of induced ovulation, with the release of endogenous gonadotropin-releasing hormone (GnRH) due to coitus. Glucocorticoid (GC) signaling, crucial for physiological homeostasis, is mediated through a yet unclear mechanism, by the GC receptor (NR3C1/GR). After mating, the female reproductive tract undergoes dynamic modifications, triggered by gene transcription, a pre-amble for fertilization and pregnancy. This study tested the hypothesis that when ovulation is induced, the expression of *NR3C1* is influenced by sperm-free seminal plasma (SP), similarly to after mating (whole semen), along the different segments of the internal reproductive tract of female rabbits. Semen (mating) was compared to vaginal infusion of sperm-free SP (*Experiment 1*), and changes over time were also evaluated, i.e., 10, 24, 36, 68, and 72 h post-mating, corresponding to specific stages, i.e., ovulation, fertilization, and the interval of early embryo development up to the morula stage (*Experiment 2*). All does were treated with GnRH to induce ovulation. Samples were retrieved from seven segments of the reproductive tract (from the cervix to infundibulum), at 20 h post-mating or sperm-free SP infusion (*Experiment 1*) or at 10, 24, 36, 68, and 72 h post-mating (*Experiment 2*). Gene expression of *NR3C1* was analyzed by qPCR. Results showed an increase in *NR3C1* expression in the infundibulum compared to the other anatomical regions in the absence of spermatozoa when sperm-free SP infusion was performed (*Experiment 1*). Moreover, during the embryo transport through the oviduct, the distal isthmus was time-course upregulated, especially at 72 h, when morulae are retained in this anatomical region, while it was downregulated in the distal uterus at 68 h (*Experiment 2*). The overall results suggest that *NR3C1*, the GC receptor gene, assessed in the reproductive tract of does for the first time, shows differential expression changes during the interval of oviductal and uterine embryo transport that may imply a relevant role of the GC action, not only close to the site of ovulation and fertilization, but also in the endometrium.

## 1. Introduction

Rabbits (*Oryctolagus cuniculus*) are wild animals originally from the south of Europe, which were domesticated and widely introduced around the world, nowadays present in every continent except Antarctica [1], and even considered as a pest in some areas [2]. These animals have been historically consumed in Mediterranean countries [3], while recently becoming important as production animals in some other regions [4]. They are also particularly well suited as model organisms for basic and applied reproductive experimental research, for their similarity to the chronology of human early embryonic development [5,6] and, especially, as a species characterized for copulation-induced ovulation as some felids [7] or camelids [8], best suited for experimental studies due to its early sexual maturation, short gestation, prolificacy and small size [9]. The use of assisted reproduction techniques (ARTs) in induced-ovulators is constrained by endocrine imbalances affecting the crucial steps of fertilization and early embryo development [10]. Therefore, the rabbit is an interesting animal model to improve intracytoplasmic sperm injection, embryo culture, embryo transfer, or cryopreservation [11], but also for commercial artificial insemination (AI) in rabbit intensive meat production [12].

Being an induced-ovulator, the rabbit requires the generation of genital-somatosensory signals during coitus to activate midbrain and brainstem noradrenergic neurons and generate the preovulatory peak of gonadotropin-releasing hormone (GnRH) [13,14]. The efficiency of natural mating, and also ARTs, relies on factors encompassing male and female parameters [15] and the interaction of both [16,17], hence improving the understanding of the effect of these factors in rabbit early development may improve fertility outcomes.

Although the role of ovarian steroid hormones progesterone and estrogens in the signaling pathways of reproductive stages is well-known, the importance of glucocorticoids (GCs) as regulators of reproduction is starting to be more recognized [18,19]. GCs are steroid hormones under the control of the hypothalamic–pituitary–adrenal axis, which are crucial for stress responses, behavior, and reproduction in mammals [20]. Even though GC production is essential for adequate physiology, they have commonly been assumed to be detrimental to reproductive performance and fertility, regarding the link between high GC levels and chronic stress [21]. However, GC basal levels have an important role in reproduction, comprising important reproductive events such as male–female mating interaction, oocyte maturation, early embryo development, fetus–mother communication, parturition, and lactation [19,22]. Although the underlying action mechanism of these hormones is complex and still not sufficiently understood, the GC receptor (NR3C1/GR) is assumed to play a key function in the mediation of GC action and gene transcription [23,24,25], including reproduction and embryo development [19,26,27,28]. The GCs bind to their receptor constituting a complex that can be transported to the nucleus, where they bind to GC response elements (GRE) of the DNA sequence, inducing the activation or repression of gene transcription [29,30], which can thereby modulate the changing female environment during the early embryo development stage [28,31,32,33]. Thus, GCs have been shown to influence the female reproductive tract, including the prostaglandin-mediated smooth muscle contractility movements [34,35], the corpus luteum formation and function [36], the Janus kinase/signal transducers and the activators of transcription (JAK/STAT) pathway, the immune response or the estrogen signaling, among others [37]. The effects of GC exposure to oocytes and preimplantational embryos are still not completely known, as whether they have a protective, innocuous, or harmful effect seems to greatly differ among mammalian species [38,39,40,41]. In rabbits, *NR3C1* has been recently postulated as a candidate gene implicated in reproductive seasonal differences between wild and domestic animals [42].

Since understanding the role of *NR3C1* in the reproductive tract is relevant for reproductive biology, we attempted to describe the GC receptor expression (*NR3C1*) of organ samples collected along the different anatomical segments of female rabbits internal reproductive tract, in response to natural mating or sperm-free seminal plasma (SP) infusion for the purpose of determining whether sperm-free SP was able to specifically affect gene expression similarly to mating, and how mating-induced *NR3C1* expression changes over time during the interval of early embryo development (10 h to 72 h).

## 2. Materials and Methods

### 2.1. Ethics Statement

The handling of the animals was performed according to the standards of animal care according to the Spanish Law (RD1201/2005), the European Directive (2010/63/EU; (BOE, 2005: 252:34367-91)) and the Directive 2010/63/EU of the European Parliament and of the Council of 22th September 2010 on the protection of animals used for scientific purposes (2010; 276:33-79). The Committee of Ethics and Animal Welfare of the Universitat Autònoma de Barcelona (Spain) approved this study (Expedient #517).

### 2.2. Animals

Adult New Zealand White rabbit bucks (*n* = 6) and does (*n* = 24), from 7 to 13 months old (mo), coming from an experimental farm of the Institut de Recerca i Tecnologia Agroalimentaries (IRTA, Torre Marimon, Spain) were used in this study. The animals were housed in individual cages for each rabbit (85 × 40 × 30 cm) equipped with plastic footrests, a feeder (restricted to 180 g/day of an all-mash pellet) and a nipple drinker (fresh water was always available). The environmental conditions were controlled, with a 16 h/8 h light/darkness photoperiod, temperature ranging from 15 to 20 °C during winter and from 20 to 26 °C during summer, and relative humidity between 60% to 75% was maintained by a forced ventilation system.

For the obtention of the ejaculate, the males were trained using an artificial vagina when they were 4.5 mo. A homemade polyvinyl chloride artificial vagina, containing water at 50 °C, was used. For this study, only one ejaculate per male was collected, discarding the ejaculates containing urine and/or calcium carbonate deposits.

### 2.3. Experimental Design

The experimental design used in this study is based on our previously described experimental approach [43].

#### 2.3.1. Experiment 1: Analysis of Gene Expression Differences in the Does’ Reproductive Tract, at 20 h Post-Mating (Whole Semen) or Seminal Plasma (SP) Infusion (Sperm-Free)

Gene expression analyses for *NR3C1* were performed in sequential segments of the female reproductive tracts (*n* = 9): endocervix (Cvx), endometrium (distal uterus: DistUt, proximal uterus: ProxUt), utero-tubal junction: UTJ, distal isthmus: Isth, ampulla: Amp, and infundibulum: Inf.

Tissue samples were collected at 20 h post-treatment with 0.03 mg GnRH im (intramuscular; Fertagyl^®^, Esteve Veterinaria, Barcelona, Spain) in all experimental groups (*n* = 9): post-mating (*n* = 3), post-SP-infusion (*n* = 3) and control, no mating or infusion, (*n* = 3, control group).

#### 2.3.2. Experiment 2: Analysis of Gene Expression Differences in the Reproductive Tract of Mated Rabbit Females from 10 h Post-mating to up to 72 h Post-mating

A group of 15 rabbit does were sequentially euthanized at 10, 24, 36, 68, and 72 h post-mating (*n* = 3, time of collection). Reproductive tract sections (Cvx, DistUt, ProxUt, UTJ, Isth, Amp, and Inf) were recovered for gene expression analysis for *NR3C1*. The 10 h post-mating group was established as the reference group as this is the presumed time of ovulation in rabbits.

### 2.4. Mating and Semen Collection

The rabbit does included in the mating groups of *Experiment 1* and *2* were sequentially mated with two randomly selected bucks to decrease male-variation effects. The does additionally received an injection of 0.03 mg GnRH im previously to being mated to reinforce ovulation. Ovulation is expected at about 10 h after GnRH stimulation in all groups.

After the semen collection from the same rabbit bucks, as described above, the sperm-free SP was isolated after centrifugation at 2000× *g* for 10 min and checked for the absence of spermatozoa. The harvested sperm-free SP was immediately pooled for vaginal infusions of *Experiment 1*.

### 2.5. Collection of Tissues and Embryos

The does were euthanized by the administration of 600 mg pentobarbital sodium (Dolethal, Vetoquinol, Madrid, Spain) intravenously (marginal ear vein). Then, the samples of the female reproductive tracts were randomly chosen from the same lateral side (right), segmented and collected. The tissues of the oviductal segments were collected in toto. In *Experiment 2*, before segment-sectioning the internal reproductive tract, the entire oviduct was isolated from the uterus. In mated does, embryos were collected by flushing the oviduct (phosphate buffer saline supplemented with 5% fetal calf serum and 1% antibiotic–antimycotic solution), which were examined by number and developmental stage. The number of ovarian follicles and the number of embryos on each developmental stage were annotated and have been published elsewhere [43]. Briefly, at 24 h, 53.0 ± 40.2% of the embryos (2-4 cell stage) were recovered, at 36 h the embryo recovery rate was 84.1 ± 31.5% (8-cell stage), at 68 h it was 103.7 ± 17.1% (morula), and at 72 h, 104.8 ± 6.7% of the embryos (compacted morula) were retrieved, with respect to the total of ovulated follicles counted at each stage (Mean ± SD). Intervals of embryo development in the mated group were extrapolated to the sperm-free SP-infused does. All reproductive segments were stored in RNAlater solution at −80 °C.

### 2.6. Real Time Quantitative PCR Analyses

The TRIzol-based protocol was used for the total RNA extraction, as described elsewhere [43]. Briefly, in 1 mL TRIzol was used to mechanically disrupt the tissues (TissueLyser II with 7 mm stainless steel beads, Qiagen, Germany). The homogenized tissues underwent different centrifugation steps and were incubated with isopropanol and RNA precipitation solution (1.2 M NaCl and 0.8 M Na_2_C_6_H_6_O_7_) for RNA pellet obtaining. The RNA concentration and quality were determined from the absorbance of 260 nm measured by Thermo Scientific NanoDrop^TM^ 2000, and the Agilent 2100 Bioanalyzer (Agilent Technologies, Palo Alto, CA, USA), respectively. The first strand cDNA synthesis was performed using the High-Capacity RNA-to-cDNA™ Kit (Applied Biosystems™, Foster City, CA, USA) and the samples were stored at −20 °C until further analyses. Quantitative PCR (qPCR) was performed in a Real-Time PCR Detection System (CFX96™; Bio-Rad Laboratories, Inc; Hercules, CA, USA) following the steps previously described [43]. Two technical replicates were used for each sample. Figure S1 depicts the melting curves and the peak curves of *β-ACTIN* and *NR3C1*. Efficiencies of the primers were calculated using five different concentrations of the same cDNA sample (serial dilutions of 1/5), using three technical replicates for each concentration. The gene relative expression levels were quantified using the Pfaffl method [44] and *β-ACTIN* as a housekeeping gene for cDNA normalization. The primer sequences, product sizes, and efficiencies are shown in Table 1. For the *β-ACTIN* gene, commercial gene-specific PCR primers for rabbit were used (PrimePCR™ SYBR^®^ Green Assay: ACTB, Rabbit; Bio-Rad Laboratories, Inc; Hercules, CA, USA). The amplicons of qPCR were loaded into an agarose gel after mixing with GelRed^®^ Nucleic Acid Gel Stain (Biotium, Fremont, CA, USA) to confirm the product sizes (Figure S2). After running, the gel was imaged by a gel imaging system (ChemiDoc XRS+ System, BioRad Laboratories, Inc; Hercules, CA, USA).

### 2.7. Statistical Analyses

All data were processed with CFX Maestro™ 1.1 software version 4.1.2433.1219 (Bio-Rad Laboratories, Inc; Hercules, CA, USA) and were analyzed for normal distribution and homoscedasticity using the Shapiro–Wilk Normality test and Levene’s test. Log(x) transformation was used to restore a normal distribution prior to analysis. The statistical analysis was conducted in R version 3.6.1. [45] with *nlme* [46] to perform linear mixed-effects (LME) models and *multcomp* [47] to perform pairwise comparisons adjusted by Tukey’s test. Data are presented as median (minimum, maximum), unless otherwise stated. The threshold for significance was set at *p* < 0.05.

Treatments of the *Experiment 1* (negative control, mating (positive control) and sperm-free SP) were used as fixed effects and each individual doe as the random part of the model. Pairwise comparisons were adjusted by Tukey’s test. A second LME model was used including the different sample collection times of *Experiment 2* (10, 24, 36, 68, and 72 h post-mating) as fixed effects and each individual doe as the random part of the model. Post-hoc comparisons were performed using Tukey’s multiple comparisons test.

Finally, the differential expression changes in qPCR results among anatomical segments (Cvx to Inf) in the *Experiment 1* and *2* were further re-analyzed, using the UTJ as an arbitrary reference anatomical medial compartment among all samples examined, which is located in the middle of the tract. This was performed in order to compare gene expression changes, per gene, issued both by mating or sperm-free SP vaginal infusion, respectively, to control (*Experiment 1*) or by different times post-mating: 10, 24, 36, 68, and 72 h (*Experiment 2*), among the different tissues of the female reproductive tract. Each tissue was included as fixed effects and each individual doe as the random part of the LME model. As stated above, the analysis of differences among each tissue of the female reproductive tract was performed by the Tukey’s multiple comparison test.

## 3. Results

### 3.1. Experiment 1: Differential Gene Expression in Rabbit Female Reproductive Tract at 20 h after Natural Mating or Infusion of Sperm-Free Seminal Plasma

**Figure 1:**
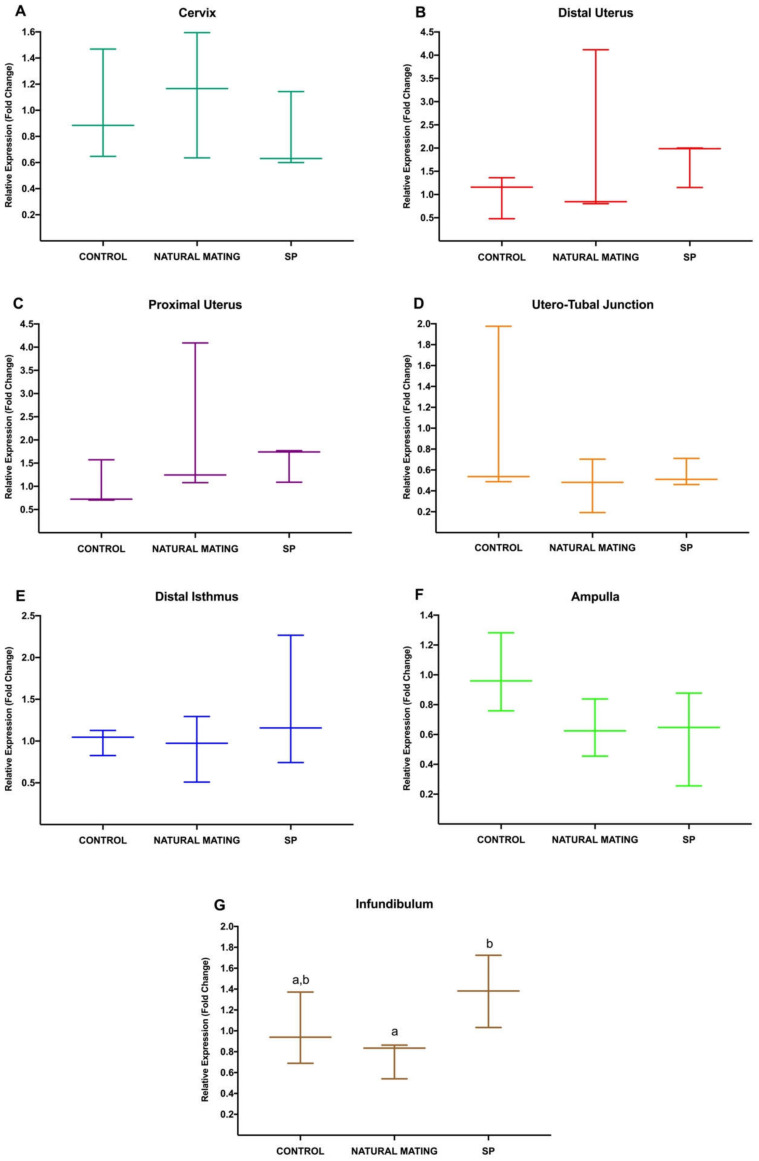
(**A**–**G**). Gene expression differences in *NR3C1* in the different anatomical segments (cervix to infundibulum; Experiment 1) (**A**–**G**), between negative control, natural mating, and seminal plasma (SP) treatments. Different letters indicate values that differed significantly between treatments in the same anatomical region (*p* < 0.05). The expression was relativized using the negative control as a reference. Median (minimum, maximum).

The results of *Experiment 1* are shown in Figure 1, where differential expression changes in *NR3C1* in the different anatomical segments of the rabbit reproductive tract were analyzed 20 h after mating or SP infusion. First, expression changes in each segment were compared between the negative control and the treatments of natural mating and SP-infusion. Significative differences in *NR3C1* expression between natural mating and SP infusion were shown in Inf (*p* < 0.05) (G), where the sperm-free SP upregulated its expression in this anatomical segment. None of the rest of the treatments displayed any significant difference (*p* > 0.05).

**Figure 2:**
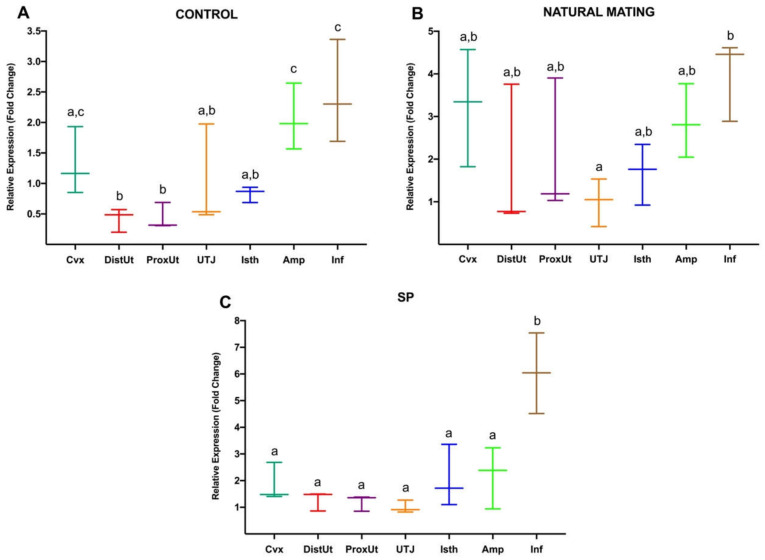
(**A**–**C**). Gene expression differences of *NR3C1* between anatomical segments (cervix, Cvx; distal uterus, DistUt; proximal uterus, ProxUt; utero-tubal junction, UTJ; distal isthmus, Isth; ampulla, Amp; infundibulum, Inf) in negative control group (**A**), natural mating (**B**), and sperm-free seminal plasma infusion (SP) (**C**). Different letters indicate values that differed significantly between anatomical regions in the same treatment (*p* < 0.05). The expression was relativized using the UTJ as a reference. Median (minimum, maximum).

Second, the expression changes triggered by each treatment were compared between anatomical regions in the doe reproductive tract, represented in Figure 2. In the negative controls (A), significant *NR3C1* upregulation was reported in Amp and Inf, relative to the rest of the anatomical segments (*p* < 0.05; except Cvx, *p* > 0.05) and also a downregulation in the uterus (DistUt and ProxUt), relative to Cvx, Amp and Inf (*p* < 0.05). In the case of sperm-free SP infusion (C), significant upregulation of *NR3C1* expression was shown in Inf compared to the rest of the anatomical segments (*p* < 0.05). Moreover, upregulation in Inf compared to UTJ was also found (*p* < 0.05) in the natural mating group.

### 3.2. Experiment 2: Differential Gene Expression in Rabbit Female Reproductive Tract from 10 h to up to 72 h in Response to Natural Mating

**Figure 3:**
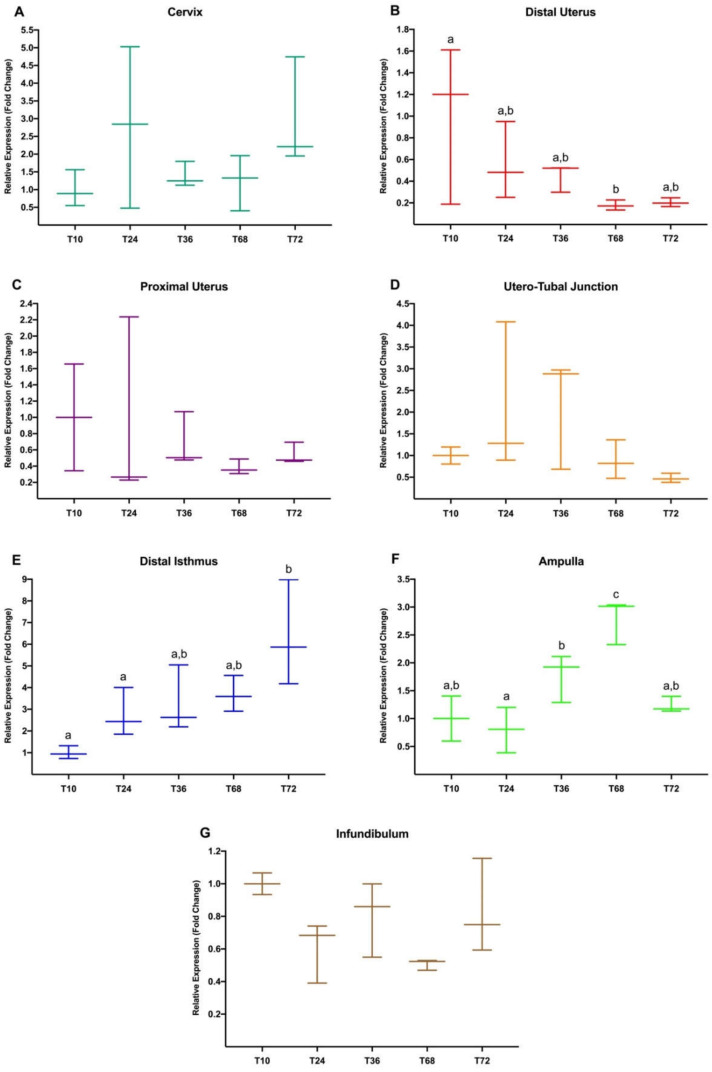
(**A**–**G**). *NR3C1* gene expression differences between each timepoint (T10, 10 h; T24, 24 h; T36, 36 h; T68, 68 h; and T72, 72 h; Experiment 2) post-natural mating in the different anatomical segments of the doe reproductive tract (cervix to infundibulum) (**A**–**G**). Different letters in the same anatomical region indicate values that differed significantly between timepoints (*p* < 0.05). The expression was relativized using the T10 as a reference. Median (minimum, maximum).

Figure 3 shows the results of *Experiment 2*, where 10 h post-mating was used to relativize the expression of the rest of the groups (24 h, 36 h, 68 h, 72 h post-mating). These timepoints are representative of embryo developmental stages. Thus, preovulatory stage, 2-4 cell embryo, 8-cell embryo, morula, and compacted morula correspond to 10, 24, 36, 68, and 72 h, respectively. In this sense, the number of ovarian follicles and embryos, and the present developmental stages, were evaluated and could be found in the aforementioned tissue collection description.

Significative differences in *NR3C1* expression were found in the DistUt (B), Isth (E), and Amp (F) at different times after mating. In the DistUt, a downregulation was found at 68 h when compared to *NR3C1* expression at 10 h post-mating (*p* < 0.05). Differently, in the Isth, *NR3C1* expression was upregulated, showing significant differences at 72 h (*p* < 0.05), when compared to 10 and 24 h post-mating. Moreover, in the Amp, the *NR3C1* gene was found to be significatively upregulated at 68h, when compared to the rest of the timepoints analyzed 10, 24, 36, and 72 h post-mating (*p* < 0.05). Upregulation at 36 h was also found when compared to 24 h in this anatomical segment (*p* < 0.05).

**Figure 4:**
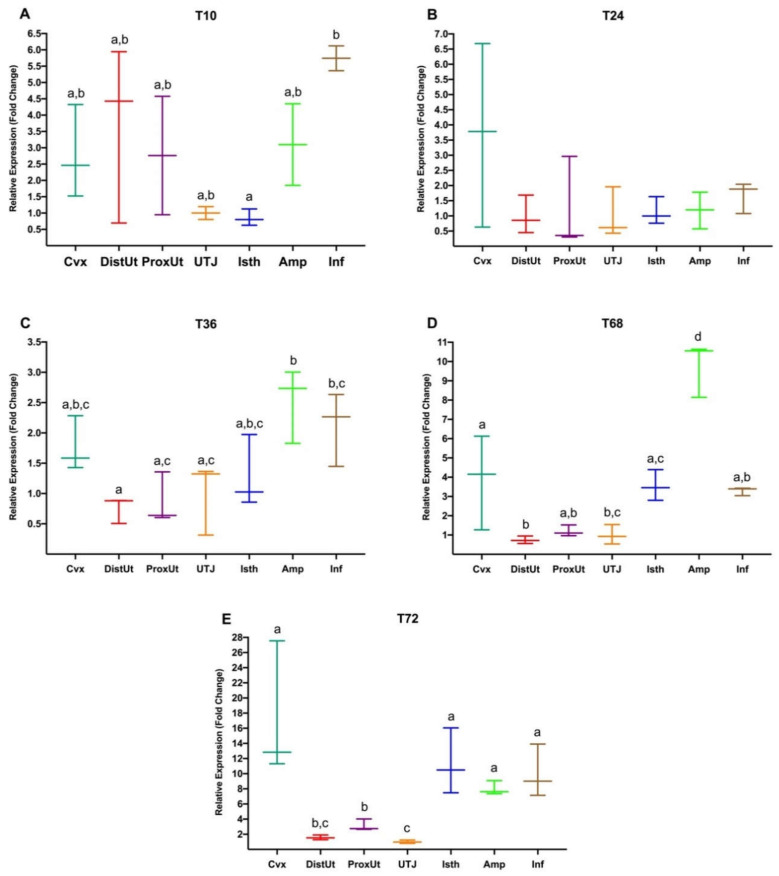
(**A**–**E**). *NR3C1* gene expression differences between anatomical segments (cervix, Cvx; distal uterus, DistUt; proximal uterus, ProxUt; utero-tubal junction, UTJ; distal isthmus, Isth; ampulla, Amp; infundibulum, Inf) in each of the timepoints 10 h (**A**, T10), 24 h (**B**, T24), 36 h (**C**, T36), 68 h (**D**, T68), and 72 h (**E**, T72) post-mating. Different letters in the same timepoint indicate values that differed significantly between anatomical regions (*p* < 0.05). The expression was relativized using the UTJ as a reference. Median [minimum, maximum].

The expression changes triggered by mating in each timepoint were also compared among the different anatomical regions (Figure 4). At 10 h post-mating (A), *NR3C1* was upregulated in the Inf compared to the Isth (*p* < 0.05). At 36 h (C), the expression of this gene was upregulated in the Amp when compared to the DistUt, ProxUt, and UTJ (*p* < 0.05) and also upregulated in the Inf, compared to the DistUt (*p* < 0.05). Similarly, after 68 h post-mating (D), significative upregulation was found in the Amp compared to the rest of the anatomical regions (*p* < 0.05). Additionally, expression in the Cvx was upregulated compared to the DistUt and UTJ (*p* < 0.05), and *NR3C1* expression in the Isth was also upregulated when compared to the DistUt (*p* < 0.05). Finally, at 72 h post-mating (E), expression was higher in the oviduct (Isth, Amp, and Inf) and also the Cvx compared to the DistUt, ProxUt, and UTJ (*p* < 0.05). Additionally, the ProxUt was upregulated compared to the UTJ (*p* < 0.05).

## 4. Discussion

In the present study, we evaluated changes in the post-ovulatory expression of the GC receptor gene (*NR3C1*) in the internal reproductive tract (cervix to infundibulum) of the female rabbit. *NR3C1* gene expression differences relative to insemination treatments and anatomical regions were analyzed 20 h after sperm-free SP infusion or natural mating, with the purpose of comparing the effects exerted by the whole semen or the sperm-free SP portion of the ejaculate (*Experiment 1*), and additionally, after mating at different timepoints (10 h, 24 h, 36 h, 68 h, and 72 h) (*Experiment 2*), during specific stages, i.e., ovulation, fertilization, and the interval of early embryo development to the morula stage achievement.

In our study, at 20 h after treatment, when ovulation may have already taken place 10 h ago [48], we found an upregulation of the *NR3C1* expression in the infundibulum, the oviductal segment where the follicular fluid and the mature oocytes are released shortly after ovulation [49]. The promotion of the receptor expression in the infundibulum was observed in both natural mated and control ovulated females, but the *NR3C1* expression was significantly higher after sperm-free SP infusion, e.g., in the absence of spermatozoa. Moreover, the upregulation in this region was significantly higher than in any other of the reproductive tract segments sampled in the study. Hence, GC receptor expression in the infundibulum seems to be generally increased after ovulation, but SP, either provided by infusion or directly by mating, may promote *NR3C1* action at this location. In this sense, SP, which is not only related to the spermatozoa transport, but also to the modulation of the immune response exerted upon sperm- free SP contact in the female reproductive tract [16], has been proven to have a positive effect on fertility in a variety of species [50], including rodents [51], pigs [52], and humans [53], and may also play a role in the GC pathway in the reproductive tract [37]. SP is produced by sexual accessory glands together with secretions from the epididymis [54] and, in rabbits, it seems to play a role in spermatozoa protection, fertilization [55] and also immune modulation during development [56]. After natural mating and sperm-free SP infusion [17], the female reproductive tract undergoes a variety of modifications that influence the initiation of controlled inflammatory response, ovulation stimulation, changes in the transcription of genes related to reproductive stages [50] and early embryonic development [57,58]. Thus, even when the SP, deposited in the cervix, does not directly reach the upper regions of the oviduct such as the infundibulum, proteins of this fluid may be absorbed by the endometrium and achieve the ovary via lymphatic route or, alternatively, by activating signaling cascades [59] that may modify the ovulation in rabbit [60]. In this way, SP molecules may modulate the overall genetic expression on different anatomical segments of the reproductive tract and, in light of our results, SP may also promote the action of GCs in the infundibulum, mediated by *NR3C1,* 20 h after GnRH injection. The specific localization of this expression change produced at this particular timepoint may be linked to the effects of SP components in the inflammatory response produced in the ovulation process. In this sense, previous studies have identified specific SP factors directly involved in the induced-ovulation triggering, such as the nerve growth factor (β-NGF). In rabbits, this mechanism of ovulation induction seems to be more complex compared to what is found in other species [8,14,60,61]. In that sense, previous studies found local effects of SP in the ovary that increased the number of hemorrhagic anovulatory follicles [62,63], suggesting an indirect action of SP on the luteinizing hormone (LH) receptors [14,64]. The differences that we found between the SP and natural mating (also containing SP) treatments, where the expression in the infundibulum seemed higher than in the rest of the tissues, although not significant, are also intriguing. In this sense, those differences between both treatments could rely on the different physical stimulation that has been reported to play an important complementary role in the rabbit ovulation, that is not required in the camelids [63]. Thus, whether the specific effect on *NR3C1* expression we observed in the infundibulum is modulated by particular SP molecules and which those molecules remains are to be elucidated, but it might be plausible that β-NGF plays a role in the GC receptor promotion that is produced at the time of the ovulation in the infundibulum.

At that moment, shortly after the LH surge, ovulation starts and the rupture of mature ovarian follicles occurs [65]. After oocytes’ release, an inflammatory-like response, very similar to other inflammatory reactions, is produced at this location [66,67], stimulated by a variety of changes in the reproductive tract including angiogenesis, vascular permeability, and exhaustive cellular differentiation, together with the production of mediators associated with inflammatory processes, such as steroids, prostaglandins, and cytokines, which may, in turn, be released to the infundibulum after the follicle rupture [65]. The GC levels can be locally regulated within the reproductive tract to serve precise functions depending on the reproductive stage temporal context [22], or the specific anatomical region [68]. Therefore, the GCs, steroid hormones well-known for their anti-inflammatory actions, are very present in the oviduct and may contribute to the phase transition by promoting healing and repair after ovulation [69]. This may also be supported by the direct detection of ten times higher total cortisol levels in the follicular fluid after the LH surge [70], together with increased levels of 11β-hydroxysteroid dehydrogenases (11β-HSD1) during the luteal phase compared to the follicular phase [71,72], responsible for the conversion of cortisone into active cortisol, which provides direct and indirect evidence of the important presence of GCs in this region.

Moreover, we found that the expression of the GC receptor is also modulated over time, corresponding to the different preimplantational embryo developmental stages, e.g., preovulatory, 2-4 cells embryo, 8-cell embryo, morula, and compacted morula that we checked, as some of the oviductal and uterine anatomical segments show very disparate expression values during the hours that were tested here (10, 24, 36, 68, and 72 h), which were only evaluated after natural mating. Here, the uterus displayed low values compared to what was found in the oviduct at 36, 68, and 72 h post-coitum. At 68 h, these uterine values were also decreased compared to the values recorded in the periovulatory phase (around 10 h), which may imply a GC action in the uterus during the period prior to embryo implantation. In this context, the widely known action of steroid hormones modulates the uterus to prepare a suitable environment for successful embryo implantation [73]. In that regard, the necessary proinflammatory effects of estradiol in the uterus, including edema, increased vascularization, and promotion of the immune antibacterial activity, have previously been shown to be antagonized by the action of GCs in different mammalian species including rat, baboon, and sheep [74,75], and may also apply to induced ovulation species, as in the case of the lagomorphs [14]. GCs have also been demonstrated to assist difficult estrogen action by blocking cell differentiation, development and growth in the uterus, which may inhibit embryo attachment [76], trophoblast invasion, and implantation [77,78,79]. However, even when high levels of GCs may be potentially detrimental for the action of estrogens, presumably predominant in the uterus at this stage, *NR3C1* uterus knockout mouse models resulted in impaired implantation, decidualization, and pregnancy [28], demonstrating that certain levels of GC regulation may be essential for an adequate uterine function.

Interestingly, an upregulation of GC action seems to be present along the rabbit oviduct at subsequent post-ovulatory timepoints according to our results. Thus, the *NR3C1* expression was found to be especially increased in the ampulla, and also in the infundibulum, at 36 h after mating and ovulation induction, when the early embryos are supposed to be transported through this oviductal region while performing the initial cell divisions (8-cell stage). At 68 h post-mating, when the embryo may be at the morula stage, the expression in the entire oviduct is also upregulated, with particularly high levels in the ampulla. This was also found at 72 h when the expression of the receptor was promoted in all the oviductal anatomical segments (distal isthmus, ampulla, and infundibulum) and also in the cervix. By this time, the morula stage is fully achieved and the embryos may have already reached the distal isthmus [80,81]. Thus, at 72 h, the levels of *NR3C1* in the distal isthmus were importantly high compared to the expression observed at the periovulatory stage (10 h after GnRH injection), and also to the expression at 24 h, shortly after fertilization is assumed to take place in upper oviductal regions [82]. This high value of *NR3C1* displayed in the distal isthmus may be explained by the previously described action of steroid hormones in smooth muscle [83]. In this tissue, where the receptor is very present [84,85], GC action may cause a decrease in the prostaglandin action [36]. Thereon, one of the functions of prostaglandins is the preimplantational embryo retention in the oviduct for approximately 3 days (72 h) by stimulating the oviductal smooth muscle contractility [34,86,87]. Moreover, RU486 (mifepristone), a glucocorticoid receptor antagonist [88], has been shown to increase the oviduct smooth muscle contractile frequency in rabbit [89]. Thus, the decrease in prostaglandins, favored by the action of steroid hormones, may be the cause of the isthmic sphincter relaxation that allows the morula embryos to enter into the uterus on its way down towards the implantation site [35]. In light of these results, GC action seems to be present along the oviduct in a distribution that may correspond to the assumed spatio-temporal location of the rabbit embryos regarding anatomical segments and timepoints, especially at the morula stage. After ovulation, the oocytes, together with follicular fluid, are released to the oviduct, where there is cross-talk between the gametes, the embryo, and the oviductal regions [90]. In this way, in vitro studies showed that cortisol production, mediated by 11β-HSD1, increased during maturation, and continued high during fertilization [91], and cortisol supplementation has improved blastocyst development rates in bovine [40], indicating that the GC activation may be part of the complex processes taken place during fertilization and early embryo development. In this sense, different results have been found regarding the effect of GCs in oocytes and early embryo development, as the influence of their presence seems to be species-specific [38]. To our knowledge, this is the first time that GC receptor levels have been described in the rabbit reproductive tract. In other species, high levels of cortisol did not affect oocyte metabolism in equine [39], while some harmful effects have been reported in pig [27,38], and different results have been shown in mice [38,92]. The levels of GC receptor are crucial in the GC regulation, however, the regulation of these hormones is complex and comprises steps that are still not completely described, involving a great number of molecules, such as 11β-HSD [38] and peptidyl-prolyl cis/trans isomerase FK506-binding proteins (i.e., FKBPs immunophilins) [93,94], among others. Thereby, the actions of GCs seem to variate among species and the importance of their regulation in reproduction is still far from being fully understood.

## 5. Conclusions

This is the first time that expression of *NR3C1*, the GC receptor gene, has been assessed in the internal reproductive tract of rabbits. Our results showed that, after sperm-free SP infusion, in the absence of spermatozoa, there is an increase in *NR3C1* expression in the infundibulum compared to natural mating. In the experiment over time, the differential expression of *NR3C1* was detected not only close to the site of ovulation and fertilization (ampulla and infundibulum), but also in the endometrium (distal uterus). The differential expressions are present over the interval during which early embryo development occurs, which may suggest a relevant role of the GC action, mediated by *NR3C1* on oviductal and uterine embryo transport. These results pave the way for further analysis that may elucidate the exact mechanism involved in the *NR3C1* action as well as its potential applications increasing the efficiency of the ARTs.

## Figures and Tables

**Table 1:** Primers used for the quantitative PCR analyses.

Gene	Primer Sequence (5′-3′)	Product Size (bp)	Efficiency (%)
*NR3C1*	F: CACAACTCACCCCAACACTG	212	89.6
	R: CAGGAGGGTCATTTGGTCAT		
*β-ACTIN*	F: commercial, not available	120	88.6
	R: commercial, not available		

*NR3C1*: glucocorticoid receptor; *β-ACTIN:* beta-actin. F: forward, R: reverse, A: adenine, C: cytosine, G: guanine, T: thymine, bp: base pair.

## Supplementary Materials

The following are available online at https://www.mdpi.com/2076-2615/10/11/2158/s1, Figure S1. Melting temperatures of PCR products of *NR3C1* (blue) and *β-ACTIN* (green) in cervix tissue (36, 68, and 72 h post-mating groups) including negative template controls (red). For DNA-binding dyes (SYBR green), the fluorescence is brightest when the two strands of DNA anneal. Therefore, as the temperature rises towards the melting temperature (Tm), relative fluorescence units (RFU) decrease at a constant rate (constant slope). At the Tm, there is a dramatic reduction in the fluorescence with a noticeable change in slope, displayed in the Melt Curve graph. The rate of this change is determined by plotting the negative first regression of fluorescence versus temperature (-d(RFU)/dT), displayed in the Melt Peak graph. The greatest rate of change in fluorescence results in visible peaks and represents the Tm of the double-stranded DNA complexes: 83.5 °C for *NR3C1*, and 89.5 °C for *β-ACTIN*. Figure S2. Agarose gel displaying PCR product size (bp: base pair) of *β-ACTIN* (120 bp) and *NR3C1* (212 bp).
