# Supplementary material for: Seminal Plasma Triggers the Differential Expression of the Glucocorticoid Receptor (NR3C1/GR) in the Rabbit Reproductive Tract
Source: Animals (Basel). 2020 Nov 19;10(11):2158. doi: 10.3390/ani10112158 (PMC7699521; doi:10.3390/ani10112158)
Supplement: Supplementary file 1 [file animals-10-02158-s001.pdf]

animals

Supplementary Materials: Seminal Plasma Triggers the Differential Expression of the Glucocorticoid Receptor (NR3C1/GR) in the Rabbit Reproductive Tract

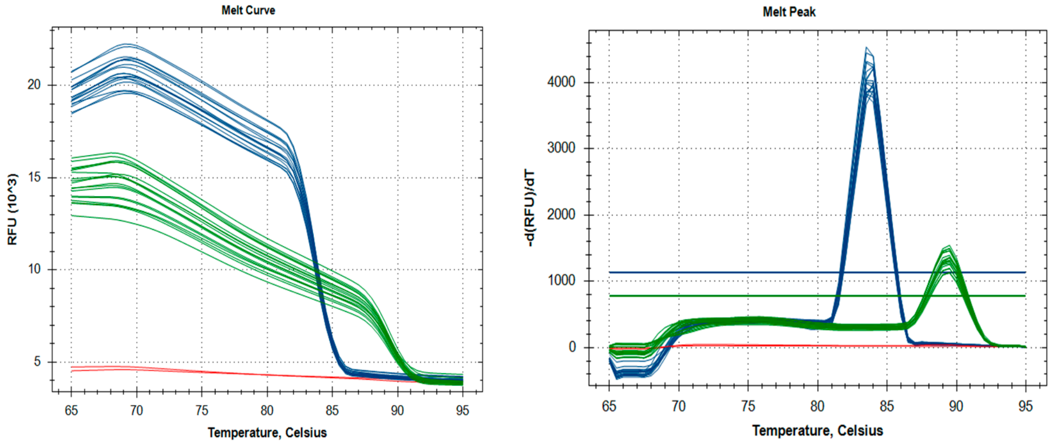

Figure S1. Melting temperatures of PCR products of *NR3C1* (blue) and *β-ACTIN* (green) in cervix tissue (36, 68, and 72 h post-mating groups) including negative template controls (red). For DNA-binding dyes (SYBR green), the fluorescence is brightest when the two strands of DNA anneal. Therefore, as the temperature rises towards the melting temperature (Tm), relative fluorescence units (RFU) decrease at a constant rate (constant slope). At the Tm there is a dramatic reduction in the fluorescence with a noticeable change in slope, displayed in the Melt Curve graph. The rate of this change is determined by plotting the negative first regression of fluorescence versus temperature (-d(RFU)/dT), displayed in the Melt Peak graph. The greatest rate of change in fluorescence results in visible peaks and represents the Tm of the double-stranded DNA complexes: 83.5ºC for *NR3C1*, and 89.5ºC for *β-ACTIN*.

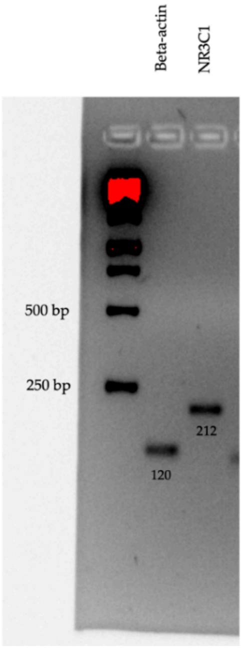

Figure S2. Agarose gel displaying PCR product size (bp: base pair) of *β-ACTIN* (120 bp) and *NR3C1* (212 bp).
